# Desmocollin-3 Is a Novel Target Receptor for Targeted Drug Delivery for Malignant Prostate Cancer

**DOI:** 10.3390/pharmaceutics18070802

**Published:** 2026-06-29

**Authors:** Vipin Sharma, Bharat Lohiya, Hanni Grace Francis, Galia Luboshits, Dror Tobi, Michael A. Firer

**Affiliations:** 1 Laboratory for Immunology and Cancer Biology, Department of Chemical Engineering, Adelson School of Medicine, Ariel University, Ariel 40700, Israel; vipins@ariel.ac.il (V.S.); bharatl@ariel.ac.il (B.L.); hannig@ariel.ac.il (H.G.F.); galialu@ariel.ac.il (G.L.); 2 Department of Molecular Biology, Ariel University, Ariel 40700, Israel; drorto@ariel.ac.il; 3 Department of Computer Science, Ariel University, Ariel 40700, Israel; 4 Adelson School of Medicine, Ariel University, Ariel 40700, Israel

**Keywords:** peptide–drug conjugates, phage display, desmocollin-3, prostate cancer, targeted drug delivery

## Abstract

**Background:** Malignant prostate cancer (PrC) remains challenging to treat due to tumor heterogeneity and the limited availability of ligands that target disease-associated surface markers with properties appropriate for targeted drug delivery systems. To overcome this hurdle, we used an unbiased but stringent selection strategy to discover a series of phage-displayed peptides that internalize specifically into PrC tumors. **Methods:** Here we report the characteristics, properties and function of one of these peptides, Pr10, and validate its ability to specifically deliver cytotoxic drugs into PrC cells and kill them, both in vitro and in xenograft models. **Results:** Biochemical and proteomic studies identified the receptor for Pr10 as Desmocollin-3 (DSC3). This finding was confirmed by demonstrating the expression of the DSC-3 protein on PrC cells; by siRNA knockdown of DSC3 expression, which abrogated Pr10 function; and by in silico docking experiments. **Conclusions:** Together, these findings identify DSC3 as a novel, functional receptor on malignant prostate cancer cells and establish Pr10 as an effective and PrC-selective ligand for drug delivery PrC cells. More broadly, this work highlights the ability of unbiased screening approaches to identify and isolate novel target receptors with properties appropriate for use in effective target drug delivery systems for cancer therapy.

## 1. Introduction

Advanced and metastatic forms of prostate cancer (PrC) remain a leading cause of cancer-related death in males [1], largely due to tumor heterogeneity and adaptive resistance mechanisms [2]. Peptide-based targeted drug delivery (pTDD) systems have emerged as a promising strategy to enhance efficacy and reduce the toxicity of cancer treatments due to their small size, ease of synthesis, lower immunogenicity, and capacity to be linked to diverse payloads [3]. In the context of prostate cancer, various targeting ligands have been explored that bind to receptors or other markers overexpressed on prostate cancer cells [4,5]. For PrC, the most notable receptor to date is prostate-specific membrane antigen (PSMA), which is exploited for the delivery of radioligand therapies and imaging agents, including ^177 Lu-PSMA-617 and its derivatives [2,6]. However, PSMA expression is heterogeneous across tumors and can be diminished in advanced prostate cancer, and PSMA-based therapy can lead to significant side effects [4,7].

PSMA was discovered serendipitously, by the immunization of mice with the PrC cell line LNCaP and isolation of a monoclonal antibody (7E11-C5) which bound a membrane protein highly expressed on these cells [8]. Importantly, such an approach does not consider the functional requirements of a receptor for the purposes of effective targeted drug delivery, which include the ability to undergo endocytosis to ensure uptake of the ligand–drug complex and intracellular release of the drug. In our previous studies on pTDD for PrC [9,10], we used a reverse strategy to identify functional targeting peptides without *a priori* knowledge of receptor identity or its natural ligand. Using phage-display peptide technology, we isolated peptides that fulfill the functional requirements of TDD systems and prepared peptide–drug conjugates (PDCs) which essentially eradicated tumor growth inhibition in xenograft models.

In the present study, we describe the isolation and identification of Desmocollin-3 (DSC3), a cadherin family protein, as a novel PrC target receptor for one of our TDD peptides, Pr10. In in silico docking experiments we located the binding site of Pr10 on the extracellular region of DSC3 and a homologous protein, N-Cadherin. These findings demonstrate that using an unbiased screening approach can uncover novel cell surface receptors that are appropriate for targeting in the selection of cytotoxic drugs to cancer cells.

## 2. Materials and Methods

### 2.1. In Vitro Binding Assay of Peptide–FITC Conjugates

PC-3 or DU-145 cells (from the ATCC) were seeded at 2 × 10^4^ cells per well in 96-well plates and cultured for 48 h at 37 °C, 5% CO_2_. The cells were washed twice with ice-cold PBS and blocked with PBS/1% BSA for 5 min on ice and incubated with FITC-labeled 7 mer cyclic peptides (see Reference [10]) at concentrations 0.1–40 µM for 10 min at 4 °C. After washing twice with ice-cold PBS, fluorescence was measured using a Tecan microplate reader (excitation/emission: 490/525 nm). Data were expressed as mean fluorescence intensity (MFI).

To investigate the binding and kinetics of peptide–FITC, three FITC-labeled peptides (PrC9-FITC, Pr10-FITC, and PrC11-FITC) were used at a final concentration of 10 µM. For the binding analysis, 1.0 × 10^5^ cells were seeded per well in 6-well plates and incubated overnight. The cells were then washed once with warm PBS and incubated with each peptide–FITC in RPMI 1640 medium at 37 °C for different time intervals (10, 20, 40, 60, and 90 min). After incubation, the cells were washed twice with ice-cold PBS, resuspended in PBS/1% BSA, and analyzed using flow cytometry (BD FACS Calibur). Fluorescence intensity was recorded in the FITC channel, and data were analyzed with FlowJo software (version v10.10.1). The percentage of FITC-positive cells was calculated relative to unstained control cells.

### 2.2. In Vitro Competition Assay

To test competitive binding between the peptides, 2 × 10^4^ cells were seeded onto microplate wells for 48 h at 37 °C at 5% CO_2_. The cells were washed twice with ice-cold PBS. FITC-labeled peptide (20 µM) was added to serially diluted unlabeled competitive peptide (200 µM to 2 × 10^−8^ µM) in a separate dilution plate for 20 min at room temperature and then the mixtures were added to the cells for 10 min at 4 °C. The cells were washed twice with ice-cold PBS and fluorescence intensity was measured in a Tecan microplate reader (excitation/emission, 490/525 nm. Nonspecific binding was assessed as the binding of a non-relevant peptide APS-3 labeled with FITC.

#### 2.2.1. Cellular Imaging of Peptide Binding in PC-3 Cells

**Confocal microscopy**—PC-3 cells were seeded on EZ-Slide chamber slides (Sigma, Rehovot, Israel). Pr10-FITC was incubated with cells for 10 min at 4 °C and then washed two times with PBS followed by fixation with 2% paraformaldehyde for 15 min at room temperature. After washing twice with PBS, nuclei were counterstained with DAPI. Imaging was carried out using a confocal microscope Zeiss LSM 7000 (Gottingen, Germany) with FITC fluorescence (green channel) used to assess peptide binding and DAPI (blue channel) to visualize the nuclei. Identical imaging parameters were applied across sample comparisons.

#### 2.2.2. Immunofluorescence Staining and Confocal Microscopy

PC-3 prostate cancer cells were seeded in confocal glass-bottom dishes. For the knockdown experiments, the cells were transfected with either negative control esiRNA cat# SIC002 or DSC3-targeting siRNA (30 nM) cat# EHU085451 for up to 48 h prior to the imaging experiments. Following transfection, the cells were incubated with FITC-labeled Pr10 peptide (Pr10-FITC) for 20 min at 37 °C. The cells were then washed twice with 1× DPBS and fixed with 4% paraformaldehyde (PFA) for 15 min at room temperature. After fixation, the cells were washed twice with Dulbecco’s PBS and incubated with 1:100 primary monoclonal cat# SC-81806 DSC3 monoclonal antibody overnight at 4 °C. The next day, the samples were washed three times with 1× DPBS and incubated with 1:500 cat# JAC-715-585-150 Alexa Fluor-conjugated secondary antibody (red channel) for 1 h at room temperature in the dark. The cells were washed twice with 1× DPBS. The nuclei were counterstained using DAPI-containing vectashield mounting medium, and fluorescence images were acquired using a Zeiss LSM 700 confocal laser scanning microscope under identical imaging settings across all conditions. Fluorescence intensity quantification was performed using ImageJ J (version 64-bit Java 8) following channel separation, as previously described. Uniform regions of interest (ROIs) were defined based on DAPI-positive nuclei, and fluorescence intensities were calculated for quantitative comparison.

#### 2.2.3. Peptide-Bound Receptor Identification

**(a)**  **Identification of peptide-bound receptor**

PC-3 cells were incubated with Pr10–biotin conjugate as described for Pr10-FITC above. After incubation, the cells were harvested and n-Octyl-β-D-glucopyranoside (OG; CAS 29836-26-8) was added at 50 mM, a concentration above its reported critical micelle concentration (CMC; ~25–30 mM), to ensure stable micelle formation and efficient solubilization of membrane-associated proteins. The lysates were centrifuged at 24,000× *g* for 20 min. and the supernatant was transferred to streptavidin-coated plates (S6940) and incubated overnight at 4 °C. The supernatant was discarded, and the wells were washed three times with PBS/0.05%Tween 20. The bound complexes were eluted using a solution of formic acid and acetonitrile (5:25, *v*/*v*), followed by incubation at 37 °C for 2 h with vigorous shaking. Eluted fractions were collected, transferred to HPLC vials, and stored at −80 °C until further processing. The samples were lyophilized overnight, reconstituted in sample buffer and resolved on Bis-Tris gels and silver-stained. Selected bands were carefully excised from the gels and submitted for LC-MS/MS and proteomic analysis (Smoler Proteomics Center, The Technion, Haifa Israel). Proteomic sequence data were analyzed to identify candidate binding proteins, with an emphasis on membrane-associated targets. Candidate confirmation was performed by repeating the streptavidin pull-down assay followed by Western blotting, where membranes were probed with specific primary monoclonal antibodies to DSC-3 (sc-81806), HSP90β (sc-517405), GAPDH (sc-365062) and PSMA (31-1214-00) and probe with secondary HRP anti-mouse IgG (715-035-150) and anti-rabbit IgG for PSMA (711-035-152) to validation of Pr10 membrane receptor identified from proteomics.

**(b)**  **Validation of peptide-bound membrane receptor**

To further evaluate the expression of DS3 of PrC cells, Western blotting was performed on PC-3 and DU145 cell lines. Cells were harvested and lysed using RIPA buffer supplemented with protease and phosphatase inhibitors. The lysates were clarified by centrifugation, and total protein concentration was determined using a BCA assay. Equal amounts of protein from each sample were denatured in loading buffer, separated on Bis-Tris polyacrylamide gels under reducing conditions, and transferred onto PVDF membranes. The membranes were blocked with 5% BSA in TBST (Tris-buffered saline with 0.1% Tween-20) for 1 h at room temperature, followed by overnight incubation at 4 °C with primary mouse monoclonal antibody of DSC-3 (sc-81806) (1:1000) and housekeeping protein GAPDH (sc-365062) (1:1000). After washing, the membranes were incubated with HRP-conjugated secondary anti-mouse (cat# 715-035-150) antibody for 1 h at room temperature. Protein bands were visualized using an enhanced chemiluminescence (ECL) detection system, and GAPDH was used as a loading control.

#### 2.2.4. Molecular Dynamics Simulations

MD simulation was performed using GROMACS 2024.2 using charmm36-jul2022 force field. Simulations were performed in a cubic box with periodic boundary conditions, with proteins located 10 Å from the box boundaries. The protein was neutralized with sodium ions. After solvent water was added with the tip3p model around the protein, and some of the water molecules were replaced with 0.15 M NaCl, energy minimization was carried out to reach the maximum force below 1000 (kJ/mol). Equilibrating the water around the protein was performed under 100 ps NVT followed by 100 ps NPT ensembles at 300 K. MD data were collected for 100 ns in the NPT ensemble at 300 K. Electrostatic interactions were calculated using the PME algorithm.

#### 2.2.5. Binding Free Energy Calculations

The Molecular mechanics/Poisson–Boltzmann surface (MMPBSA) area binding free energy between the peptide and the protein during the MD simulation was calculated using the package gmx_MMPBSA with the protein–protein binding free energy protocol.

#### 2.2.6. Cytotoxicity Analysis of Peptide–Drug Conjugate Pr10-MMAE

PC-3 and DU-145 cells as well as human lung fibroblasts (HFLs) and HaCat cells (from the ATCC) were seeded into 96-well white opaque plates (3–5 × 10^3^ cells/well) and allowed to attach for 24 h before treatment. Cytotoxicity was assessed by exposing the cells to serial dilutions of Pr10-MMAE in triplicate for 30 min, after which the drug-containing medium was replaced with fresh medium and incubation continued for a total of 72 h. Cell viability was then measured using the CellTiter-Glo^®^ luminescent cell viability assay following the manufacturer’s protocol, and luminescence was recorded on a Tecan microplate reader. Data were normalized to the untreated controls and analyzed in GraphPad Prism 10.6.0 using nonlinear regression to calculate IC_50_ values.

#### 2.2.7. esiRNA-Mediated Silencing of DSC3 and Quantitative Real-Time PCR

Gene silencing was performed using esiRNA targeting Desmocollin-3 (DSC3), while a non-targeting esiRNA served as a negative control. PC3 cells were transfected according to the manufacturer’s recommendations and incubated for 48 h to allow efficient knockdown prior to RNA extraction. Total RNA was isolated using a commercial RNA isolation kit (QuantaBio, Beverly, MA, USA) following the manufacturer’s protocol, and RNA concentration and purity were assessed spectrophotometrically. Equal amounts of RNA were reverse-transcribed into cDNA using the qScript™ cDNA Synthesis Kit (QuantaBio; Cat# 95047100). Quantitative real-time PCR (qRT-PCR) was performed using PerfeCTa^®^ SYBR^®^ Green FastMix Low ROX (QuantaBio; Cat# 95074012) on a real-time PCR system according to the manufacturer’s instructions. DSC3 expression was amplified using the following primers: DSC3-1A (forward) CACAGAAGCACCTGGAGACG; and DSC3-1B (reverse) ATGCAATTTTTCACCGAGACGG, synthesized as desalted oligonucleotides (0.02 µmol scale). β-actin (forward) AGGTCTTTGCGGATGTCCACGT and (reverse) CACCATTGGCAATGA GCGGTTC was used as the endogenous reference gene for normalization. Primer specificity and amplification efficiency were validated prior to quantitative analysis. Amplification specificity was confirmed by melt-curve analysis. Relative DSC3 mRNA expression levels were calculated using the 2^−ΔΔCt^ method, normalized to β-actin, and expressed relative to the control cells. All reactions were performed in technical triplicates, and data are presented as relative fold change. DSC3 knockdown efficiency confirmed at the mRNA level was used to support downstream functional analyses evaluating Pr10 binding under silencing conditions.

#### 2.2.8. Animal Studies

Athymic male nu/nu mice 6–8 weeks of age were purchased from Envigo Labs (Jerusalem, Israel) and housed under sterile conditions. All animal experiments were performed according to protocol Au-IL-2508-112 prepared according to ARRIVE 2.0 guidelines and approved by the Ariel University Animal Ethics Committee.

#### 2.2.9. Anti-Tumor Effect of Pr10 MMAE PDCs

A xenograft tumor model was established by injecting mice subcutaneously with 1.5 × 10^6^ logarithmic PC3 cells into the right flank region with Matrigel. Tumor growth was monitored regularly, and the mice were randomized into one of four groups (*n* = 5 per group) when their tumor volume reached approximately 140 mm^3^ (approx. day 16 post-injection) to ensure the equal distribution of tumor burden among the groups. The mice were treated twice weekly for two weeks via tail vein injection as follows. Group 1—control vehicle only; Group 2 received free MMAE at a dose of 0.8 mg/kg; Group 3 received Pr10-MMAE at a dose of 1.5 mg/kg; Group 4 received Pr10-MMAE at a dose of 3 mg/kg. Tumor volume and body weight were measured regularly with a caliper until day 49 from the initiation of treatment after which all the mice were euthanized via CO_2_ inhalation. The mice whose tumors reached 600 mm^3^ were removed from the trial.

## 3. Results

### 3.1. Binding Properties of Phage-Derived Peptides in Prostate Cancer Cells

**Figure 1:**
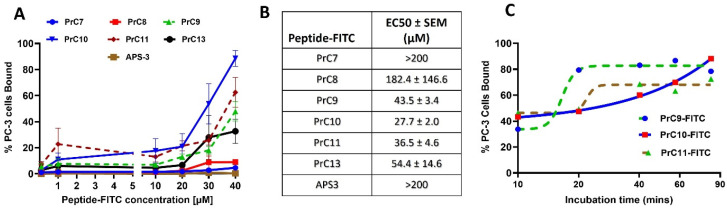
**Binding characteristics of prostate cancer-binding peptides.** (**A**) Concentration-dependent binding of FITC-labeled peptides to PC-3 cells measured by spectrofluorometric fluorescence polarization at 4 °C. Data are presented as percentage peptide binding normalized as per mean fluorescence intensity (MFI) of each peptide for 3 repeat experiments. (**B**) EC50 values derived from the graphs shown in A. (**C**) Time-dependent binding of FITC-labeled PrC9, Pr10, and PrC11 to PC-3 cells measured by flow cytometry at 37 °C. Data are presented as percentage of peptide-stained positive cells over time.

We recently reported the use of a phage-display peptide library to isolate a series of 7 mer cyclic peptides that specifically bind to and internalize into PrC cells [10]. To further characterize their suitability for pTDD, Fluorescein Isothiocyanate (FITC) conjugates of these peptides and a non-relevant cyclic 7 mer (APS-3) were shown to differ in their binding strengths to PC-3 cells at 4 °C (Figure 1A). Peptide Pr10 exhibited the lowest EC50 (27.71 ± 2.01 µM), amongst these peptides (Figure 1B). PrC11 (36.51 ± 4.63 µM) and PrC9 (43.52 ± 3.39 µM) also showed relatively low EC50 values, while PrC7 and APS-3 displayed EC50 values in the millimolar range, consistent with poor binding. To compare the binding kinetics of the three peptides with the lowest IC50, the respective peptide–FITC conjugates of PrC9, Pr10 and PrC11 were incubated with PC-3 cells for 10, 20, 40, 60 and 90 min and binding was assessed by flow cytometry. Figure 1C shows that PrC9 binding becomes saturated earlier than the other two peptides. Pr10 has a more linear binding behavior. These results suggest that PrC9 and −10 may target different receptors.

**Figure 2:**
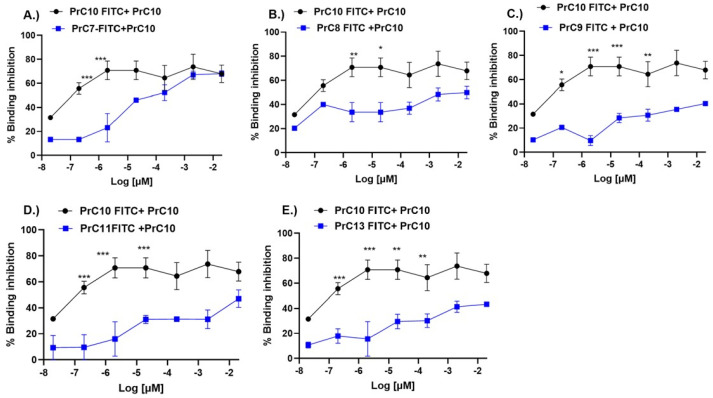
**Competitive binding of five PrC-specific peptides**. Unlabeled Pr10 displaced the FITC peptide almost completely, with a dissociation constant (Kd) of 3.09 × 10^−8^ ± 1.09 M and a maximum binding (Bmax) of 70.53 ± 1.87%, confirming strong and specific binding. In the presence of PrC7 (**A**), the Kd increased to 6.29 × 10^−6^ ± 2.18 M, while Bmax remained largely unchanged at 68.78 ± 2.27%, indicating competitive binding with reduced affinity but preserved maximal displacement. PrC8 (**B**) showed a Kd of 3.73 × 10^−8^ ± 3.70 M and reduced Bmax to 47.40 ± 2.41%, suggesting partial competition with significant loss of binding capacity (pIC_50_ = 2.84 ± 1.08). PrC9 (**C**) and PrC11 (**D**) show less binding inhibition, with Kd values of 1.03 × 10^−5^ ± 5.64 × 10^−6^ M and 7.30 × 10^−6^ ± 5.25 M, respectively, and Bmax values decreased to 42.49 ± 2.48% (PrC9) and 47.21 ± 3.49% (PrC11). PrC13 (**E**) showed a similar pattern with Kd = 6.95 × 10^−6^ ± 4.61 M and Bmax reduced to 44.74 ± 2.97% (pIC_50_ = 4.41 ± 1.84). Data points represent mean +/− SEM for at least three repeat experiments. *, *p* < 0.05; **, *p* < 0.001; ***, *p* < 0.0001.

To test this possibility, competitive displacement assays were performed using FITC-labeled Pr10-FITC and Pr10 as the standard and increasing concentrations of unlabeled peptides as competitors (Figure 2A–E). Unlabeled Pr10 efficiently displaced Pr10-FITC in a dose-dependent manner, confirming specific and saturable binding. PrC7, PrC8, and PrC13 only partially reduced Pr10-FITC binding, whereas PrC9 and PrC11 showed only weak inhibition even at high concentrations. These results indicate that although multiple peptides bind PC-3 cells, only a subset interact with binding sites that overlap with Pr10, while PrC9 and PrC11 are likely to recognize different surface targets. From these assays, affinity parameters were calculated (Table 1). Pr10 demonstrated the most robust binding, efficient self-displacement, and stable performance across assays; Pr10 was selected for subsequent receptor identification and functional studies.

The stability of the PDC-MMA was tested by measuring the % MMAE release after incubation at pH 5.0 and 7.4 buffers or in normal human serum for 25 h. As shown in Figure S2, the PDC was completely stable at pH7.4 and 82% intact in human serum after 25 h.

### 3.2. In Vivo Studies

**Figure 3:**
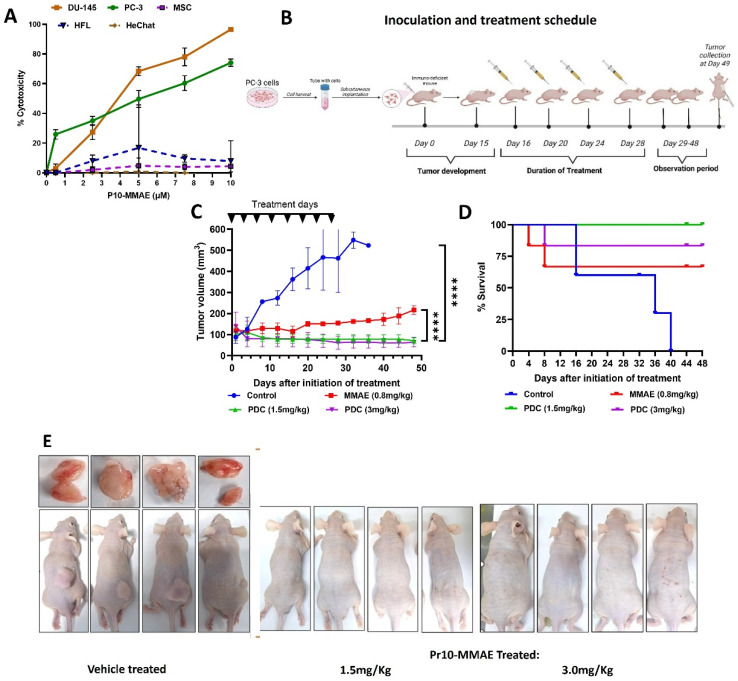
**In vitro and in vivo cytotoxic efficacy of Pr10-MMAE PDC.** (**A**) The dose–response cytotoxicity of the Pr10-MMAE PDC. PrC cell lines and normal cells were exposed to increasing concentrations (0.1–10 µM) of Pr10-MMAE, washed and then cultured without PDC for 48 h before cell viability was measured. PrC cells—PC-3, Du-145; non-cancerous cell lines—HFL (human lung fibroblast), MSC (Human Mesenchymal Stem Cells) and HeCAT (immortalized human keratinocyte cell line). (**B**) An outline of the PrC-xenograft model used to test the efficacy of Pr10-MMAE PDC therapy. (**C**) The time-course of tumor growth in PC3-tumor-bearing mice treated on the designated days (▼) with either vehicle (blue), free MMAE (0.8 mg/kg) (red), or Pr10-MMAE PDC at a dose of either 1.5 mg/Kg (green) or 3 mg/Kg (purple). (**D**) The Kaplan–Meir survival curves of the mice from the xenograft trial. (**E**) Images of PC-3-bearing mice: Controls—images taken after sacrifice on the day tumor volume exceeded the maximum limit of 600 mm^3^. Tumor position is outlined. Tumors were excised and are shown at the top. PDC-treated mice—images taken on day 48 and the close of the experiment **** *p* < 0.0001.

In our recent study describing the discovery of mPrC-targeting peptides [10], we demonstrated that Pr10 selectively labeled PC-3 xenograft tumors but not normal prostate cells or other organs such as the heart or spleen. A PDC was then synthesized by conjugating Pr10 to the anti-mitotic drug Monomethyl auristatin E (Pr10-MMAE). Figure 3A demonstrates that the PDC was highly toxic to the PrC cell lines with a calculated EC50 of 6.5 µM (95% confidence limits 0.3216–0.6041, triplicates) for PC-3 cells and 4.2 µM (95% confidence limits 1.206–2.973, triplicates) for DU-145 cells. In contrast, reactivity against normal HFL and HaCat cells was negligible even at the highest PDC dose tested (10 µM).

The therapeutic efficacy of Pr10-MMAE was tested in a subcutaneous PC-3 xenograft model (Figure 3B). Animals received two doses per week for four weeks of either vehicle (PBS, control), free MMAE (0.8 mg/kg), or Pr10-MMAE at 1.5 or 3 mg/kg doses on the days indicated and were then monitored for a further 20 days for tumor volume and signs of morbidity. In the control mice tumors grew aggressively, reaching the maximum tolerable size in nearly 50% of the group by day 32 post-cell-inoculation and in 100% by day 56 (Figure 3C,D (left)). Treatment with free MMAE initially retarded tumor growth, but tumor volume increased soon after cessation of treatment. In addition, 30% of the mice in this group died during the treatment period (Figure 3D). In contrast, both doses of Pr10-MMAE completely halted tumor growth and average tumor volumes in both groups declined, even after the first treatment. In the 1.5 mg/kg group none of the animals showed signs of systemic toxicity; in the 3 mg/kg group, one animal died from reasons not related to drug toxicity (Figure 3D). Statistical analysis confirmed a highly significant efficacy of Pr10-MMAE treatment compared to both the control and free-MMAE-treated groups (*p* < 0.0001).

### 3.3. Isolation and Identification of Pr10-Binding Receptor

**Figure 4:**
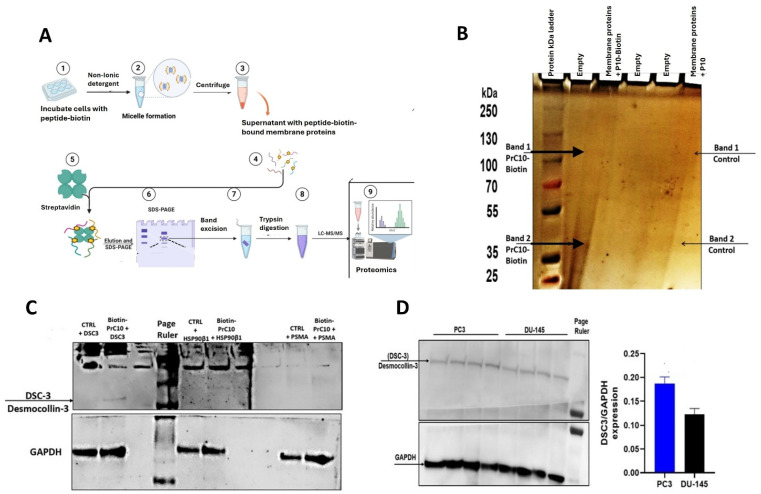

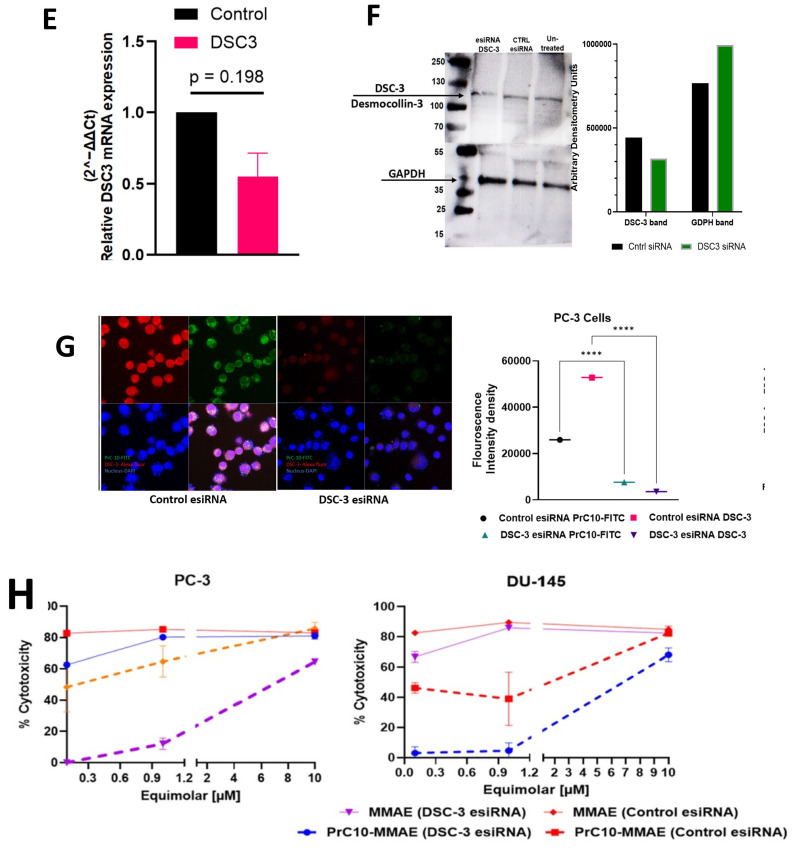
**Identification of Pr10-binding membrane proteins.** (**A**): A schematic of the experimental workflow used to isolate Pr10-membrane-binding proteins from PC-3 cells. (**B**): Bis-Tris-4–12% Page gel showng Pr10–biotin-enriched protein bands (Band 1 ~100 kDa and Band 2 ~35 kDa). (**C**): PC-3 cell lysates were incubated with biotin-labeled Pr10 peptide or control peptide, followed by streptavidin pull-down and immunoblotting. A distinct band corresponding to Desmocollin-3 (DSC3) was detected in the biotin-Pr10 lane. Control experiments using HSP90β1 and PSMA antibodies showed no enrichment. (**D**): Western blot analysis comparing DSC3 protein expression between PC-3 and DU-145 prostate cancer cell lines. The quantification of DSC3 normalized to GAPDH confirmed significantly elevated DSC3 expression in the PC-3 cells (*p* < 0.05). (**E**): RT–qPCR analysis of DSC3 expression in PC3 cells transfected with control esiRNA or DSC3-targeting esiRNA. Relative expression levels were calculated using the 2^−ΔΔCt^ method and normalized to β-actin. Data are presented as mean ± SEM from three independent biological replicates. Statistical analysis was performed on the ΔCt values using an unpaired two-tailed *t* test (*p* = 0.198). (**F**): Western blot validation of DSC3 protein knockdown in PC-3 cells treated with DSC3-specific esiRNA displayed reduced DSC3 protein levels compared with the untreated and control esiRNA groups. Densitometric analysis of the DSC3/GAPDH ratios confirmed decreased DSC3 expression after knockdown (*p* < 0.0001, two-way Chi-Square). (**G**): Confocal fluorescence microscopy demonstrating receptor-dependent peptide binding. PC-3 cells were incubated with FITC-labeled Pr10 peptide (green) and stained with DSC3 antibody (Alexa Fluor, red) with DAPI for nuclear staining (blue). DSC3 knockdown markedly reduced Pr10-FITC binding and DSC-Alexa Fluor fluorescence signal. The quantification of fluorescence intensity from confocal imaging experiments. DSC3 knockdown significantly reduced peptide binding intensity. Statistical analysis indicated a highly significant reduction in peptide binding following DSC3 silencing (**** *p* < 0.0001). (**H**) Functional cytotoxicity assays evaluating the activity of the Pr10-MMAE peptide–drug conjugate compared with free MMAE in PC-3 and DU-145 cells.

We previously reported that Pr10 does not bind PSMA on PrC cells [10]. To identify the Pr10 receptor, we developed a peptide-based affinity pull-down approach outlined in Figure 4A. Cells were incubated with Pr10–biotin (step 1), lysed (steps 2,3), and peptide–membrane protein complexes captured using streptavidin-coated microplate wells (steps 4,5). The bound proteins were eluted, separated on Bis-Tris-PAGE and silver-stained (step 6), which revealed two bands only seen in the Pr10–biotin lane (Figure 4B) at approximately ~100 kDa (Band 1) and ~35 kDa (Band 2). The two Pr10-enriched bands in lane 3 as well as the corresponding gel locations in lane 6 were excised and analyzed by LC-MS/MS (steps 7–9). Band amino acid sequences were run through the Smoler Proteomics Center’s workflow protocol as described in Materials and Methods to identify candidate proteins. The peptide sequences identified by MS were found in 1919 proteins. By filtering these proteins according to the ratio of abundance of the recovered sequence in the higher molecular weight band in +Pr10 lane (lane 3) over the abundance of that sequence in the corresponding control lane (lane 6), 15 proteins were identified as possible Pr10 binding candidates (see the file in Supplementary Material). Of these proteins, Desmocollin-3 (DSC3) is a membrane-bound protein and Hsp90 has been reported to be membrane-bound under certain conditions [11]. To study these candidates further, we first repeated the affinity isolation procedure up to step 6, and then performed immunoblotting. This showed enrichment of DSC3 in the Pr10–biotin samples compared with the control sample (Figure 4C). In contrast, no enrichment was observed for unrelated membrane proteins such as HSP90β or PSMA, demonstrating the specificity of the interaction. GAPDH served as a loading control. We next examined DSC3 expression in PC-3 and DU-145 prostate cancer cells. Western blot analysis revealed DSC3 expression in both cell lines, with higher levels observed in the PC-3 cells compared with the DU-145 cells (Figure 4D and Figure S3).

### 3.4. siRNA Knockdown Validation of DSC3 as the Pr10-Binding Receptor

To validate the direct connection between DSC3 expression and Pr10 function, we first performed RT-qPCR analysis following esiRNA transfection in PC3 cells. Relative expression levels were calculated using the 2^−ΔΔCt^ method with β-actin as an internal reference. DSC3 mRNA levels were reduced to approximately 0.55-fold relative to the control DSC3RNA-treated cells, corresponding to an estimated ~50% decrease in transcript levels (Figure 4E). Densitometric analysis of the DSC-3 and control GAPDH Western blot bands confirmed a significant reduction in DSC3 protein expression in DSC3-esiRNA-treated cells compared with the control esiRNA and untreated cells (Figure 4F, *p* < 0.0001, two-way Chi-Square 46042, df = 1, z = 214.6).

DSC3 siRNA knockdown also reduced the functional uptake of Pr10 uptake into the PC3 cells. Confocal imaging (Figure 4G) showed that cells treated with control esiRNA stained strongly with anti-DSC3-Alexa Fluor (red) and readily internalized Pr10-FITC (green), with clear overlap observed in the merged images (pink). In contrast, DSC3 siRNA treatment resulted in a striking reduction in DSC3 fluorescence expression and no peptide uptake. Quantitative Image J analysis confirmed a significant reduction in DSC3 fluorescence following knockdown compared with the control cells (Figure 4G). These results were supported by flow cytometry (Figure S4). Finally, PC-3 and DU-145 cells knocked down with either DSC3siRNA or control esiRNA were cultured for 48 h with increasing concentrations of Pr10-MMAE PDC. Figure 4H shows that receptor knockdown reduced the cytotoxicity of 1 µM PDC from 60% to 10% in the PC-3 cells and from 40% to 5% in the DU-145 cells. These results provide direct functional evidence that the identified DSC-3 receptor is necessary for efficient Pr10 function as a drug carrier.

### 3.5. In Silico Desmocollin-Pr10 Docking

**Figure 5:**
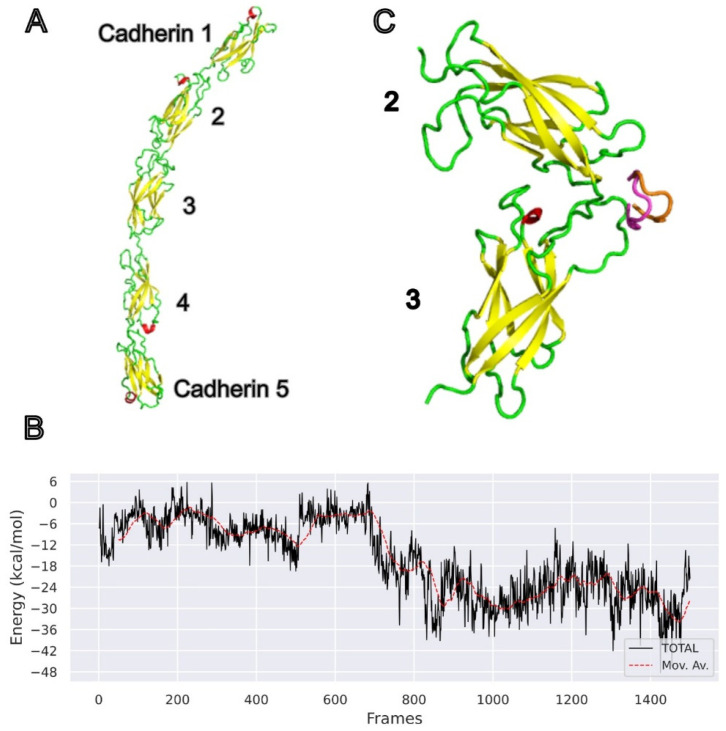
**The docking of the peptide to Desmocollin-3.** (**A**) An Alphaford3 model of the extracellular domain of Desmocollin-3. The protein is shown in a cartoon representation, colored by secondary structure (helices red, sheets yellow, and loops green) (**B**). The binding ∆G energy of the peptide. (**C**) The peptide binding poses at 125 ns (orange) and 150 ns (magenta) to the cleft between Cadherin 2 and 3 of Desmocollin-3.

An alphafod3 model was created for the extracellular domain of Desmocollin-3 (Uniprot Q14574, amino acids 136–668), comprising the domains Cadherin 1–5 and Pr10 (Figure 5A). Docking of the peptide was performed with two additional servers, LZerD and HPEPDOCK. The LZerD Web Server predicted that all the top five Pr10 models bind to the Cadherin2 domain. Alphafold3 predicted four models to bind to Cadherin 1 and one model to Cadherin 2. The HPEPDOCK predicted two models to bind to Cadherin2, one to Cadherin1, one to Cadherin4, and one to the cleft connecting Cadherin 2 and 3. These models were rescored using Prodigy (Explosion AI, Berlin, Germany), and the top three with the lowest binding energy (two from LZerD and one from HPEPDOCK) showed binding to Cadherin2. These models were subjected to a 10 ns MD simulation, and the pose with the best MMPBSA binding ∆G was then subjected to a 100 ns simulation. The simulation shows that the peptide does not bind to a specific site but rather samples multiple binding poses along the lower part of Cadherin2, close to the connecting cleft with Cadherin3. Energetically, two binding states were identified binding ∆G of −20.9 kcal/mol and–9.2 kcal/mol (see Figure S5A). Therefore, we performed local docking in this region using Rosetta. All five top models were docked to the cleft between Cadherin 2 and 3. Using the top docked model, a 150 ns simulation was conducted. The MMPBSA binding ∆G between the peptide and DSC3 is depicted in Figure 5B. After 70 ns of equilibration or induced-fit, the binding energy is settled around −25.8 kcal/mol based on averaging the last 60 ns. Throughout the simulation, the peptide remains bound in the cleft. Figure 5C presents Cadherin 2 and 3 colored by secondary structure, with the peptide binding poses at 125 ns (orange) and 150 ns (magenta). The two poses are in proximity.

Local sequence alignment of the region forming Cadherin2–3 of Desmocollin-3 and Cadherin-2 (Uniprot P19022, alternative name N-cadherin) was performed using the EMBL-EBI Job Dispatcher water algorithm (see Supplementary Figure S6). The two sequences show 39.2% sequence identity, suggesting that the peptide may bind to Cadherin-2 as well. A model of the peptide bound to the cleft between the domains of Cadherin-2, Cadherin 2 and 3, was generated by comparative modeling with Modeler. A 100 ns MD simulation was performed, and the peptide remained bound to the cleft throughout the run. The last frame of the simulation is shown in Figure S7A; Cadherin domains 2 and 3 of Cadherin-2 are colored by secondary structure, and the bound peptide is colored in magenta. The binding ∆G of the peptide along the run is shown in Figure S7B with an average ∆G = −29.96 kcal/mol, indicating that the binding to Cadherin-2 might be similar or better than to Desmocollin-3.

## 4. Discussion

The availability of PDCs that selectively internalize into and specifically kill malignant PrC cells may represent a breakthrough event in providing a much-improved prognosis for patients with malignant prostate cancer. A crucial step towards developing effective pTDD systems is to identify cell surface components on the target cell by their ability to effectively and selectively deliver the PDC to the intracellular machinery, rather than by their overexpression on the target cell [12]. To achieve this goal for malignant PrC, we used one of our recently reported PrC-targeting peptides, Pr10 [10], and an unbiased screening approach and proteomics to identify DSC3 as a novel PrC cell receptor (Figure 4). There is very little published information on the expression of DSC3 protein expression in advanced prostate cancer. Pan et al. reported the downregulation of DSC3 mRNA in advanced prostate cancer; however, they did not measure DSC3 protein levels [13]. Our results indicate that protein expression can persist even when mRNA transcript abundance decreases. Indeed, despite repeated attempts at calibration of the esiRNA protocol, we could not eliminate DSC3 expression completely (Figure 4F). Moreover, our proteomic data also identified the presence of PCBP1 (Supplementary—Proteomic receptor candidate), a protein reported to bind mRNA and enhance its translation [14]. Whether or not PCBPI is involved in maintaining low-level expression of DSC3 mRNA remains to be studied.

The proteomic data also identified the involvement of Cofilin-1 (CFL1), a key mediator of cytoskeletal remodeling during epithelial–mesenchymal transition [15], in Pr10 binding, suggesting that peptide binding occurs within a broader adhesion cytoskeletal complex. In fact, PC3 cells also express high N-cadherin protein expression [14], and we found by in silico docking that Pr10 also binds CADH2 (N-cadherin) (Figure S5), and furthermore, that the DSC3-associated cadherin 2 subunit (Figure 5A–C) has 58.6% sequence similarity (Figure S6). This domain forms part of a structurally conserved interface shared across the cadherin superfamily, including N-cadherin [16,17], suggesting the peptide targets a common cadherin surface in PC-3 cells.

Although DSC3 is not exclusively restricted to malignant cells (The Human Protein Atlas, https://www.proteinatlas.org/ENSG00000134762-DSC3 (accessed on 26 May 2026), differences in protein accessibility, membrane organization, and endocytic activity between tumor and normal tissues may provide a membrane-associated environment that favors the selective binding of Pr10 to PrC cells, an additional level of selectivity that is not captured by transcriptomic analyses alone [13,18].

## 5. Conclusions

Several potential non-PSMA biomarkers are currently undergoing pre-clinical evaluation for mPrC theranostics, including hormone receptors, immune-checkpoint-related glycoproteins, intracellular proteins, and tumor microenvironment proteins [19,20]. Most of these candidates are being targeted with known ligands labeled with radioisotopes and were selected for their relative overexpression in or on mPrC cells rather than for their suitability for drug delivery systems. Our findings provide a clear framework for the discovery and validation of new surface targets using peptide-based approaches. By integrating functional screening of phage display-derived peptide followed by surface receptor identification, we demonstrate that novel surface targets can be uncovered without prior knowledge of their identity Our data establish DSC3 as a novel, exploitable surface target and provide the first in vivo proof-of-concept for peptide-mediated DSC3 targeting as a therapeutic strategy for targeted drug delivery to PrC cells.

## Figures and Tables

**Table 1:** Comparative potency (pIC50) and maximal binding (Bmax and Kd) of different peptides on PC3 cells.

Peptide	Kd #	% Bmax ##	pIC50
**Pr10-FITC + Pr10**	3.09 × 10^−8^ ± 1.09	70.53 ± 1.87	7.51 ± 0.15
**PrC7-FITC+ Pr10**	6.29 × 10^−6^ ± 2.18	68.78 ± 2.27	5.20 ± 0.15
**PrC8-FITC+ Pr10**	3.73 × 10^−8^ ± 3.70	47.40 ± 2.41	7.43 ± 0.04
**PrC9-FITC+ Pr10**	1.03 × 10^−5^ ± 5.64 × 10^−6^	42.49 ± 2.48	4.99 ± 0.24
**PrC11-FITC+ Pr10**	7.30 × 10^−6^ ± 5.25	47.21 ± 3.49	5.14 ± 0.31
**PrC13-FITC + Pr10**	6.95 × 10^−6^ ± 4.61	44.74 ± 2.97	5.16 ± 0.29

#—Kd (dissociation constant); ##—% Bmax (maximum specific binding inhibition).

## Data Availability

The data created in this study are available by contacting the corresponding author.

## Supplementary Materials

The following supporting information can be downloaded at: https://www.mdpi.com/article/10.3390/pharmaceutics18070802/s1, Figure S1: Time-dependent binding of FITC-labeled peptides (PrC9, PrC10, and PrC11) to PC-3 cells; Figure S2: The stability of PDC-MMAE was tested at pH 5.0, pH 7.4, and human serum. Figure S3: Flow cytometry analysis of PC-3 cells unstained (Left) or stained with Anti-Desmocollin-3 antibodies; Figure S4: Flow cytometry analysis of AntiPrC10-FITC peptide binding following DSC3 knockdown; Figure S5: Molecular dynamics simulation of PrC10 to Cadherin 1; Figure S6: Local sequence alignment of the region forming the domains Cadherin2 and 3 between Desmocollin-3 (DSC3_HUMAN) and Cadherin-2 (CADH2_HUMAN) Cadherin2; Figure S7: PrC10 peptide binding to Cadherin-2. (A) The last frame of the MD simulation of Cadherin domains 2 and 3 of Cadherin-2 with the peptide.

## Abbreviations

The following abbreviations are used in this manuscript:

DSC3	Desmocollin-3
pTDD	Peptide-based targeted drug delivery
PDC	Peptide–drug conjugate
OG	n-Octyl-β-D-glucopyranoside
