# Supplementary material for: Desmocollin-3 Is a Novel Target Receptor for Targeted Drug Delivery for Malignant Prostate Cancer
Source: Pharmaceutics. 2026 Jun 29;18(7):802. doi: 10.3390/pharmaceutics18070802 (PMC13415596; doi:10.3390/pharmaceutics18070802)
Supplement: Supplementary file 1 [file pharmaceutics-18-00802-s001.zip › pharmaceutics-4372626-supplementary.pdf]

Desmacolin-3 is a novel target receptor for Targeted Drug Delivery for Malignant Prostate Cancer

SUPPLEMENTARY Information

Time-dependent binding of FITC-labeled peptides to PC-3 cells.

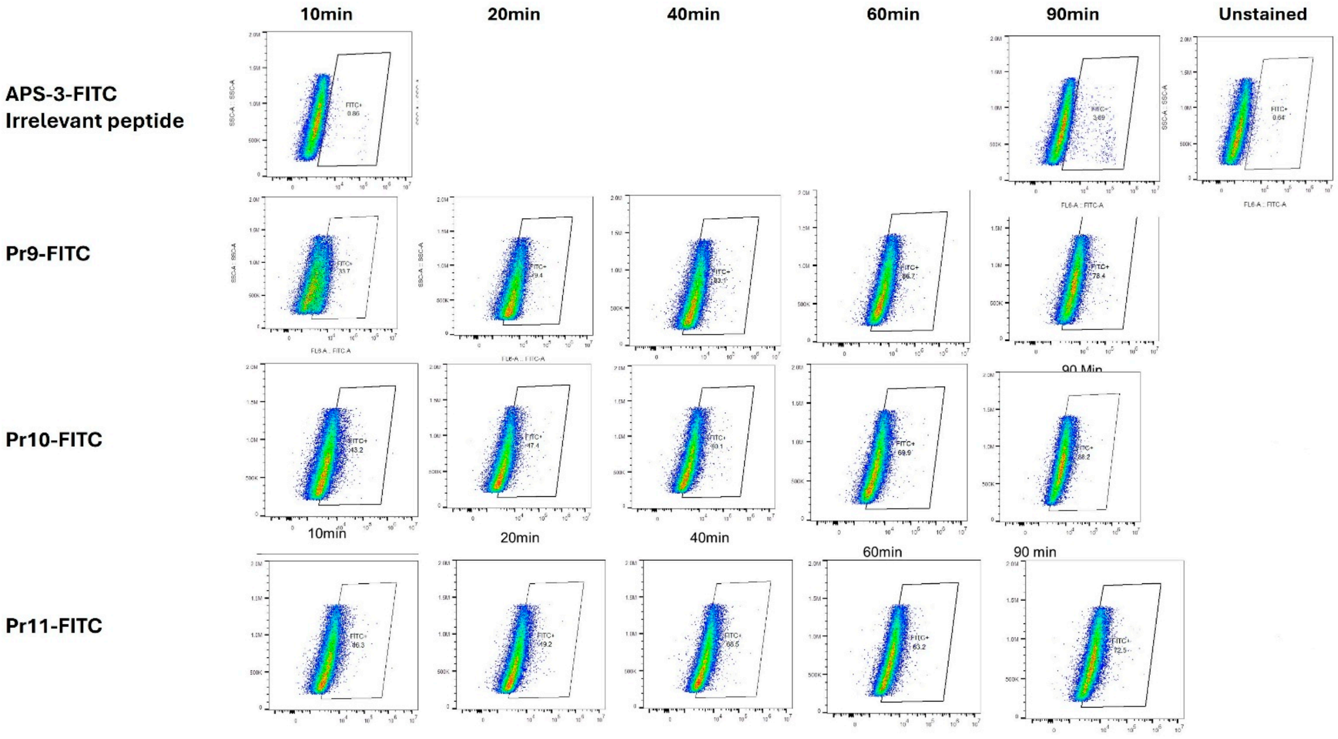

Figure S1: Time-dependent binding of FITC-labeled peptides (PrC9, PrC10, and PrC11) to PC-3 cells. *Flow cytometry analysis was performed to measure binding of 10 µM FITC-labeled peptides at 37 °C in different incubation times. PrC9-FITC showed rapid binding, with more than 80% of cells positive within 20 minutes and reaching saturation shortly after. PrC10-FITC displayed slower but steady binding, with uptake increasing gradually and reaching nearly 80% of cells after 90 minutes. PrC11-FITC showed moderate binding, reaching about 60-65% of cells between 40 and 60 minutes without full saturation. Unstained cells and APS-3 FITC-treated cells at 10 min and 90 min were included as controls to confirm minimal background fluorescence and specificity of peptide binding.*

PDC-MMAE stability

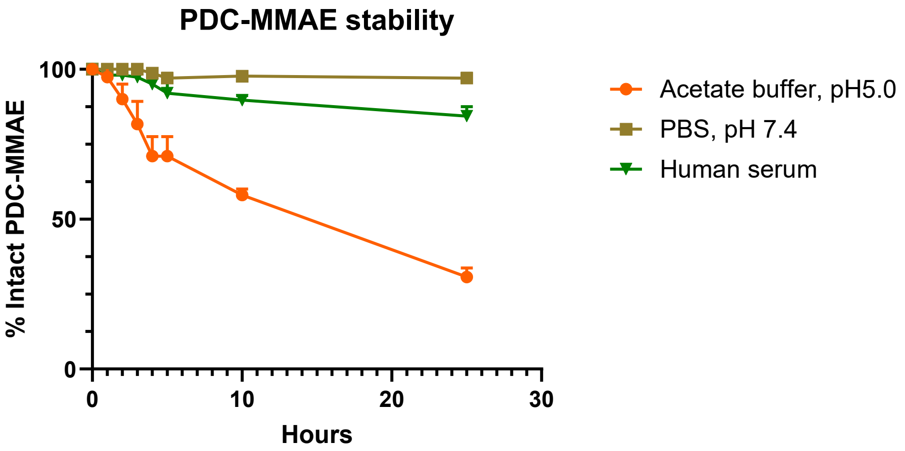

Figure S2: The stability of PDC-MMAE was tested at pH 5.0, pH 7.4, and human serum. Ten µM of PDC were added to each of the solutions and filtered through syringe into HPLC vials. The mixtures were kept at room temperature. At the time intervals shown on the graph, samples were taken, centrifuged at 14,000g for 15 min at 4°C, the supernatant was filtered and analyzed using High Resolution Mass Spectrometry (Waters Micro mass Quattro Micro instrument). The PDC remained essentially intact in neutral buffer (PBS pH 7.4), whereas acidic conditions (acetate buffer, pH 5) enhanced degradation. In human serum, the PDC as 82% intact after 25hrs incubation.

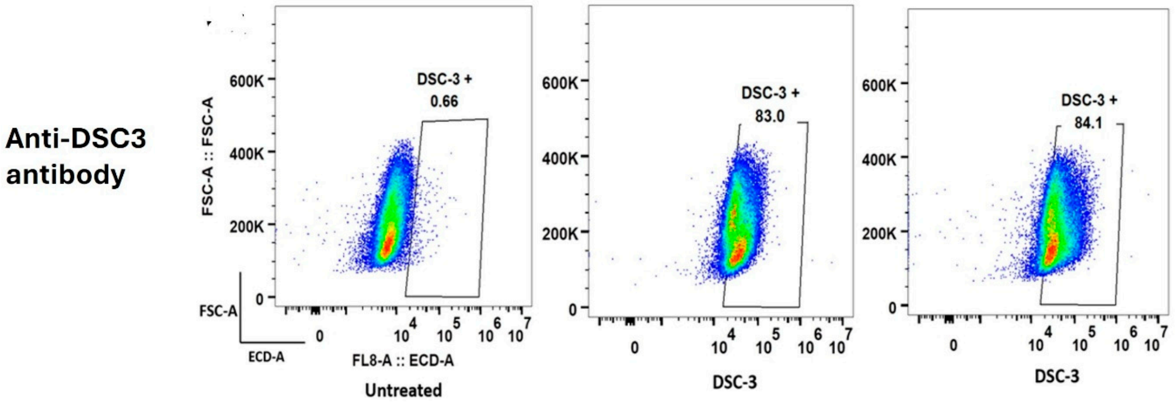

Figure S3: Flow cytometry analysis of PC-3 cells unstained (Left) or stained with Anti-Desmocollin-3 antibodies (middle and left).

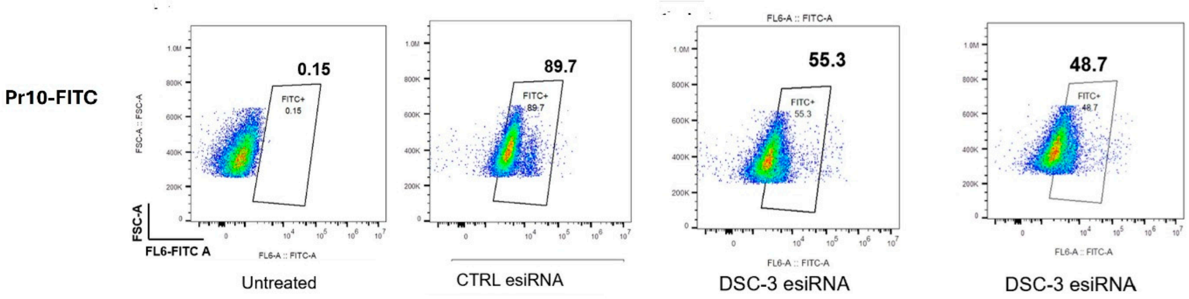

Figure S4. *Flow cytometry analysis of AntiPrC10-FITC peptide binding following DSC3 knockdown. Control esiRNA cells showed high peptide binding 89.7% FITC-positive cells. In contrast, DSC3 knockdown significantly reduced peptide binding 55.3% and 48.7% demonstrating receptor-dependent peptide interaction.*

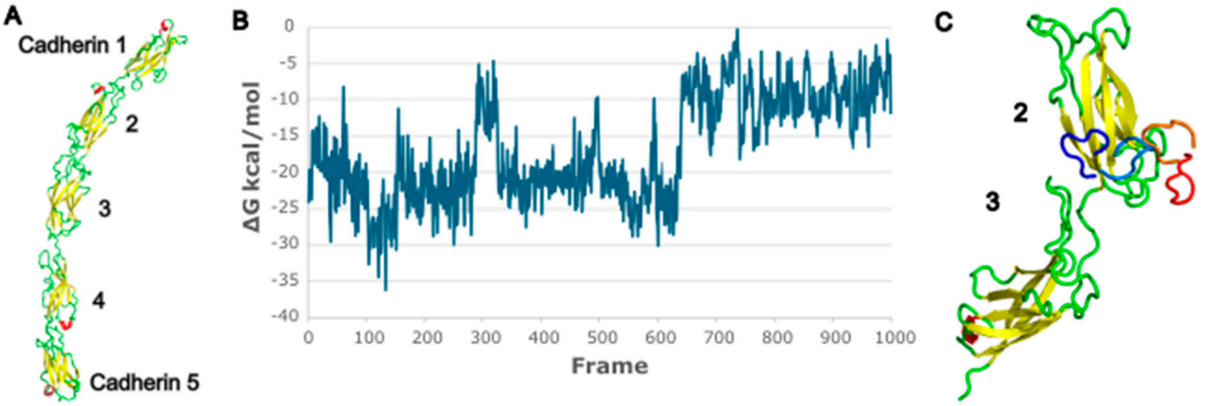

***Figure S5: Molecular dynamics simulation of PrC10 to Cadherin 1**. (A) Alphaford3 model of the extracellular domain of Desmocollin-3. The protein is shown in a cartoon representation, colored by secondary structure (helices red, sheets yellow, and loops green) (B). The binding ΔG energy of the peptide to the Cadherin2 domain of Desmocollin-3. The MD simulation shows two modes of binding. One, during the first 63 ns with an average binding ΔG of -20.9 kcal/mol, and the second, during the last 37 ns with an average ΔG of -9.2 kcal/mol. (C) Four randomly selected binding poses. The PrC10 peptide is shown using a cartoon representation. Two binding poses from the first phase (lower energy) are colored red and orange, and two from the second phase (higher binding energy) are colored cyan and blue. Cadherin domains 2 and 3 colored by secondary structure.*

```
# Aligned_sequences: 2
# 1: DSC3_HUMAN
# 2: CADH2_HUMAN
# Matrix: EBLOSUM62
# Gap_penalty: 10.0
# Extend_penalty: 0.5
#
# Length: 237
# Identity:      93/237 (39.2%)
# Similarity:   139/237 (58.6%)
# Gaps:          10/237 ( 4.2%)
# Score: 434.0
#
#
#=======================================

DSC3_HUMAN       237 NDNHPVFTEAIYNFEVLESSRPGTTVGVVCATDRDEPDTMHTRLKYSILQ    286
                     |||.|.|...::|..|.|.|:|||.|..|.|.|.|:|:.::..|:|.|:.
CADH2_HUMAN      261 NDNRPEFLHQVWNGTVPEGSKPGTYVMTVTAIDADDPNALNGMLRYRIVS    310

DSC3_HUMAN       287 QTPR--SPGLFSVHPSTGVITTVSHYLDREVVDKYSLIMKVQDMDGQ-FF    333
                     |.|.  ||.:|:::..||.|.||:..||||.|.:|:||::..||:|. .:
CADH2_HUMAN      311 QAPSTPSPNMFTINNETGDIITVAAGLDREKVQQYTLIIQATDMEGNPTY    360

DSC3_HUMAN       334 GLIGTSTCIITVTDSNDNAPTFRQNAYEAFVEENAFNVEILRIPIEDKDL    383
                     ||..|:|.:|||||.|||.|.|....:...|.||..::.:..:.:.|||.
CADH2_HUMAN      361 GLSNTATAVITVTDVNDNPPEFTAMTFYGEVPENRVDIIVANLTVTDKDQ    410

DSC3_HUMAN       384 INTANWRVNFTILKGNENGHFKISTDKETNEGVLSVVKPLNYEENRQVNL    433
                     .:|..|...:.|..|:..|.|.|.||..:|:|:::||||:::|.||...|
CADH2_HUMAN      411 PHTPAWNAVYRISGGDPTGRFAIQTDPNSNDGLVTVVKPIDFETNRMFVL    460

DSC3_HUMAN       434 EIGVNNEAPFARDI---PRVTALNRALVTVHVRDLDE    467
                     .:...|:.|.|:.|   |:.|    |.|:|.|.|::|
CADH2_HUMAN      461 TVAAENQVPLAKGIQHPPQST----ATVSVTVIDVNE    493
```

***Figure S6**: Local sequence alignment of the region forming the domains Cadherin2 and 3 between Desmocollin-3 (DSC3_HUMAN) and Cadherin-2 (CADH2_HUMAN) Cadherin2.* *The two sequences show 39.2% sequence identity and 58.6% sequence similarity. The alignment was performed using the water algorithm of EMBL-EBI Job Dispatcher*

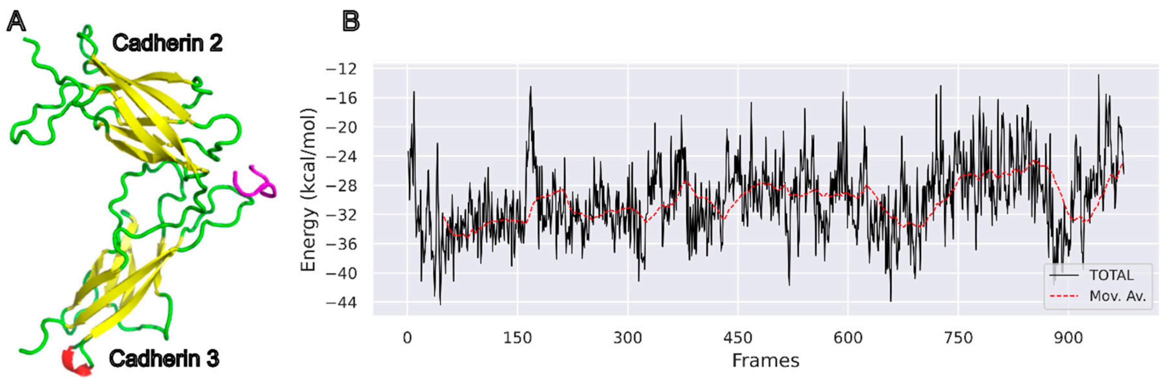

Figure S7: *PrC10 peptide binding to Cadherin-2. (A) The last frame of the MD simulation of Cadherin domains 2 and 3 of Cadherin-2 with the peptide. The protein is shown in a cartoon representation, colored by secondary structure (helices red, sheets yellow, and loops green) and the peptide is colored magenta. (B). The binding ΔG energy of the peptide during the simulation.*
